# The Anaortic Technique with Bilateral Internal Thoracic Artery Grafting - Filling the Gap in Coronary Artery Bypass Surgery

**DOI:** 10.21470/1678-9741-2020-0451

**Published:** 2021

**Authors:** Walter J. Gomes, Eduardo N. Gomes, Ayrton Bertini Jr, Pedro H. Reis, Nelson A. Hossne Jr

**Affiliations:** 1 Cardiovascular Surgery Discipline, Hospital São Paulo, Escola Paulista de Medicina, Universidade Federal de São Paulo, São Paulo, Brazil.; 2 Affiliated Hospitals of Associação Paulista para o Desenvolvimento da Medicina (SPDM), São Paulo, São Paulo, Brazil.

**Keywords:** Coronary Artery Bypass, Atrial Fibrilation, Coronary Artery Diseases, Plaque Atherosclerotic, Stroke, Aorta, Embolism

## Abstract

Coronary artery bypass grafting (CABG) has consolidated its role as the most effective procedure for treating patients with advanced atherosclerotic coronary artery disease, reducing the long-term risk of myocardial infarction and death compared to other therapies and relieving angina. Despite the recognized benefits afforded by surgical myocardial revascularization, a subset of higher-risk patients bears a more elevated risk of perioperative stroke. Stroke remains the drawback of conventional CABG and has been strongly linked to aortic manipulation (cannulation, cross-clamping, and side-biting clamping for the performance of proximal aortic anastomoses) and the use of cardiopulmonary bypass. Adoption of off-pump CABG (OPCAB) is demonstrated to lower the risk of perioperative stroke, as well as reducing the risk of short-term mortality, renal failure, atrial fibrillation, bleeding, and length of intensive care unit stay. However, increased risk persists owing to the need for the tangential ascending aorta clamping to construct the proximal anastomosis. The concept of anaortic (aorta no-touch) OPCAB (anOPCAB) stems from eliminating ascending aorta manipulation, virtually abolishing the risk of embolism caused by aortic wall debris into the brain circulation. The adoption of anOPCAB has been shown to further decrease the risk of postoperative stroke, especially in higher-risk patients, entailing a step forward and a refinement of outcomes provided by the primeval OPCAB technique. Therefore, anOPCAB has been the recommended technique in patients with cerebrovascular disease and/or calciﬁcation or atheromatous plaque in the ascending aorta and should be preferred in patients with high-risk factors for neurological damage and stroke.

**Table:** 

Abbreviations, acronyms & symbols				
**anOPCAB**	**= Anaortic (aorta no-touch) OPCAB**		**MI**	**= Myocardial infarction**
**ART**	**= Arterial Revascularization Trial**		**MRI**	**= Magnetic resonance imaging**
**BITA**	**= Bilateral internal thoracic artery**		**NT**	**= No-touch**
**CABG**	**= Coronary artery bypass grafting**		**OM**	**= Obtuse marginal**
**CAD**	**= Coronary artery disease**		**ONCAB**	**= On-pump CABG**
**CPB**	**= Cardiopulmonary bypass**		**OPCAB**	**= Off-pump CABG**
**CI**	**= Confidence interval**		**PCI**	**= Percutaneous coronary intervention**
**Dg**	**= Diagonal artery**		**PDA**	**= Posterior descending coronary artery**
**DSWI**	**= Deep sternal wound infections**		**RA**	**= Radial artery**
**ESC/EACTS**	**= European Society of Cardiology/European** **Association for Cardio-Thoracic Surgery**		**RCA**	**= Right coronary artery**
			**RITA**	**= Right internal thoracic artery**
**GOPCABE**	**= German Off-Pump Coronary Artery Bypass** **Grafting in Elderly Patients**		**SBI**	**= Silent brain infarcts**
			**SITA**	**= Single internal thoracic artery**
**ITA**	**= Internal thoracic artery**		**SV**	**= Saphenous vein**
**LAD**	**= Left anterior descending coronary artery**		**SVG**	**= Saphenous vein graft**
**LITA**	**= Left internal thoracic artery**			

## INTRODUCTION

Endorsed by recent and robust evidence, coronary artery bypass grafting (CABG) has solidified its role as the most effective procedure for treating patients with advanced atherosclerotic coronary artery disease (CAD), reducing the long-term risk of myocardial infarction (MI) and death compared to other therapies, besides relieving angina^[1-5]^.

Despite the recognized benefits afforded by surgical revascularization, a subset of higher-risk patients bears a more elevated risk of perioperative neurological complications, and stroke remains the drawback of conventional CABG. Data from administrative databases and observational registries suggest that the incidence of perioperative stroke after cardiac surgery ranges from 0.8% to 5.2%^[6]^.

The occurrence of CABG perioperative stroke displays a bimodal configuration. Approximately half of the perioperative strokes are identified immediately on the patient's awakening from anesthesia (intraoperative/early strokes), resulting mainly from manipulation of the aorta with cerebral atheroembolization or the release of particulate matter from the cardiopulmonary bypass (CPB) circuit. The other half occurs days after an initial uneventful recovery and is defined as postoperative/delayed strokes. Delayed strokes are often consequent to postoperative atrial fibrillation, perioperative MI with intracavitary thrombus formation, or previous cerebrovascular disease. Both early and delayed strokes are related to a significant increase in the postoperative period as well as late morbidity and mortality, however, early stroke is associated with a significantly higher operative mortality than delayed stroke, a 12-fold increase (29% *vs*. 2% without stroke)^[7,8]^.

## TACKLING PERIOPERATIVE NEUROLOGICAL COMPLICATIONS AND STROKE

Neurological complications comprising stroke, delirium, and cognitive decline associated with on-pump CABG (ONCAB) have been strongly linked to aortic manipulation (cannulation, cross-clamping, and side-biting clamping for the performance of proximal aortic anastomoses) and use of CPB. Early stroke is usually located in the cerebral right hemisphere, consistent with the jet of the flow from the aortic cannula^[6]^.

Adoption of off-pump CABG (OPCAB) and avoidance of aortic manipulation lower the risk of perioperative stroke^[9]^. The elimination of CPB decreases not only the risk of stroke, but also the short-term mortality, renal failure, atrial fibrillation, bleeding, and length of intensive care unit stay^[9]^. The use of OPCAB has broadened the indication of surgical revascularization for patients at high risk for undergoing CPB, including the very elderly and patients with impending end-organ failure. Although OPCAB significantly reduces the incidence of postoperative neurological complications compared to ONCAB^[10,11]^, increased risk persists owing to the need for the tangential ascending aorta clamping to construct the proximal anastomosis^[12]^.

The concept of anaortic (aorta no-touch) OPCAB (anOPCAB) has emerged as a viable and effective technical solution for patients at high risk for perioperative neurological damage or stroke. This concept stems from eliminating ascending aorta manipulation, virtually abolishing the risk of embolism caused by aortic wall debris into the brain circulation^[9,13]^. The adoption of anOPCAB further decreases the risk of postoperative stroke, especially in higher-risk patients, with reported rates at < 0.4%^[9,12]^, entailing a step forward and a refinement on outcomes provided by the primeval OPCAB technique. Therefore, anOPCAB has been the recommended technique in patients with cerebrovascular disease and/or calciﬁcation or atheromatous plaque in the ascending aorta and should be preferred in patients with high-risk factors for neurological damage and stroke^[9-17]^.

## HIGH-RISK PATIENTS FOR PERIOPERATIVE STROKE

Regardless of the severity of aortic atherosclerotic involvement, clamping the aorta during CABG increases the risk of postoperative stroke^[14]^. However, certain subgroups of patients face an enhanced risk for perioperative neurological injury. The known risk factors associated with neurological injury after CABG encompasses advanced age, aortic atheromatous disease, aortic manipulation, diabetes, female sex, hypertension, peripheral vascular disease, previous neurological injury, symptomatic carotid stenosis, and use of CPB^[18]^. Age is one of the most significant predictors of brain injury and the risk for perioperative stroke is 4.6 times higher for individuals 65 to 75 years old and 5.2 times higher for patients over 75 years of age, compared with those under 65 years^[6]^. Aorta with wall thickness > 4 mm and aortic plaques protruding > 3 mm increase the risk of adverse neurologic outcomes during aortic manipulation^[19,20]^.

The higher rate of stroke following CABG is found in the first 30 days after the procedure, subsequently the incidence of stroke in a 31-day to five-year follow-up is similar between CABG and percutaneous coronary intervention (PCI)^[21]^. Therefore, limiting, if not completely eliminating, aortic manipulation performing an anOPCAB procedure substantially reduces stroke rates^[9,14,21-24]^. In octogenarians, a contemporary meta-analysis of studies comparing outcomes of ONCAB and OPCAB revealed that OPCAB provided lower in-hospital mortality, stroke rate, and length of hospital stay with a similar incidence of other adverse outcomes. Preferentially offering OPCAB to octogenarians could be translated into a reduced economic burden on the healthcare providers^[25]^. In diabetic patients, who currently comprise nearly half of patients referred for CABG^[26]^, individual patient-data pooled analysis demonstrates that five-year stroke rates nearly doubled after CABG compared with PCI^[21]^.

## THE EMERGING ISSUE OF SILENT BRAIN INFARCT

Silent brain infarcts (SBI) are clinically silent, neuroimaging-diagnosed infarcts, and although the initial insult is not clinically apparent, SBI has been linked to significant later morbidity. The risk of subsequent stroke increases more than five times when SBI is present, which may reflect associated underlying risks and additional associated sequelae including cognitive dysfunction, increased risk of dementia, psychiatric disturbances, and reduced quality of life^[27]^. The real incidence of postoperative SBI may be markedly higher than the clinically evident strokes as shown on magnetic resonance imaging (MRI) after CABG and surgical aortic valve replacement^[28,29]^. Postoperative diffusion-weighted MRI reveals that new brain infarcts after CABG are significantly more frequent than clinically evident stroke, reaching 27.6% of the operated patients, most of the lesions being clinically silent^[30]^, stressing that becomes imperative the introduction of strategies to minimize this incidence^[31]^.

## THE RATIONALE FOR ANAORTIC OPCAB WITH BILATERAL INTERNAL THORACIC ARTERY GRAFTING

**Fig. 1:**
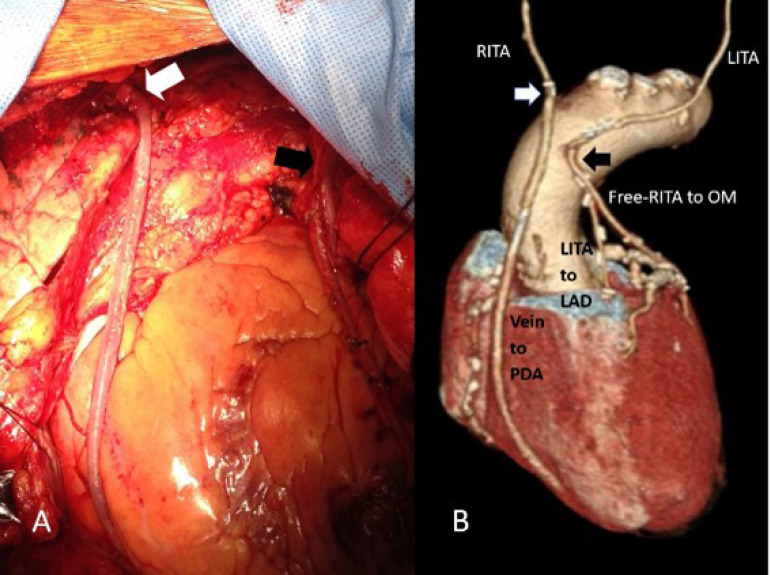
A. Proximal anastomosis of the free right internal thoracic artery (RITA) graft to the left internal thoracic artery (LITA) in a Y-fashion (black arrow); saphenous vein graft (SVG) to posterior descending coronary artery (PDA) proximally grafted end-to-end to the RITA stump (white arrow). B. Postoperative computed tomography coronary angiography depicting the end-to-end SVG-RITA stump anastomosis (white arrow) and the free RITA graft proximal anastomosis to LITA (black arrow). LAD=left anterior descending coronary artery; OM=obtuse marginal.

**Fig. 2:**
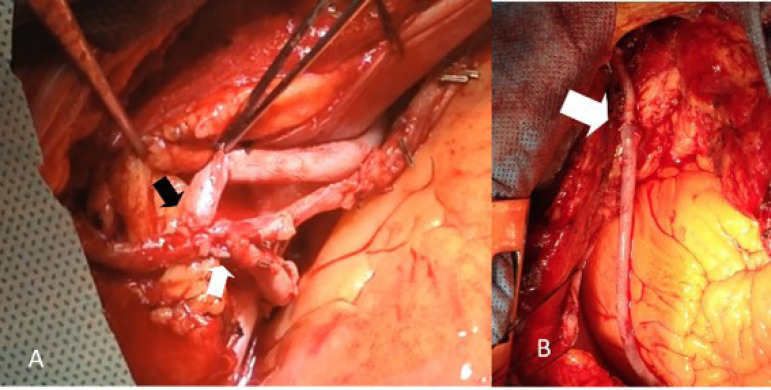
A. The free right internal thoracic artery (RITA) to obtuse marginal (white arrow) and saphenous vein graft to diagonal (black arrow) proximally anastomosed to the left internal thoracic artery to left anterior descending artery in a Y-configuration. B. Vein graft to posterior descending coronary artery proximally anastomosed to the RITA stump (white arrow).

**Fig. 3:**
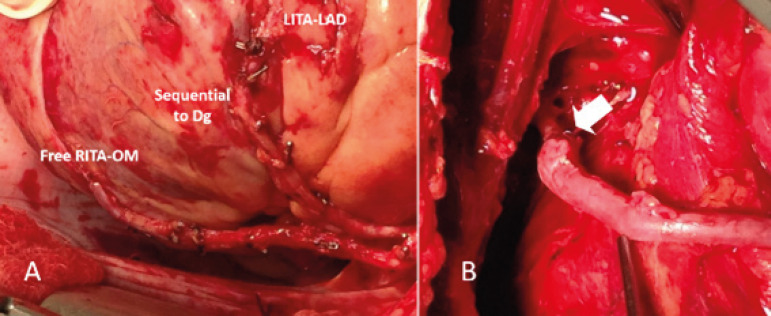
A. Left internal thoracic artery (LITA) sequentially grafted to left anterior descending coronary artery (LAD) and diagonal. Free right internal thoracic artery (RITA) to obtuse marginal (OM) connected to LITA in a Y-fashion. B. The saphenous vein graft to posterior descending coronary artery sutured end-to-end to the RITA stump (white arrow). Dg=diagonal artery.

The utilization of bilateral internal thoracic artery (BITA) grafts is paramount for fulfilling the concept of anOPCAB, supplemented with the saphenous vein (SV) or radial artery (RA) as a Y-composite graft based on the in situ skeletonized BITA, enabling complete coronary revascularization (Figures 1, 2, and 3).

**Fig. 4:**
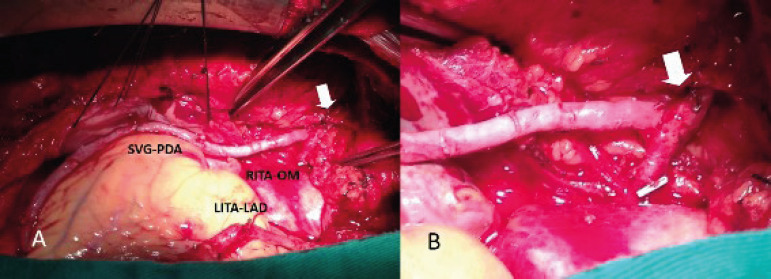
A. In situ right internal thoracic artery (RITA) grafted to obtuse marginal (OM) and routed through the transverse sinus. The saphenous vein graft (SVG) to posterior descending coronary artery (PDA) is proximally anastomosed end-to-side to RITA (white arrow). B. Detail of the SVG end-to-side anastomosis to RITA stump (white arrow). LAD=left anterior descending coronary artery; LITA=left internal thoracic artery.

Similar midterm patency rates, improvement of myocardial perfusion, and clinical outcomes between vein grafts and arterial grafts are reported when veins are used as a composite graft based on the internal thoracic artery (ITA)^[32]^. In the SAVE RITA trial, a randomized trial comparing the strategy of using the right ITA (RITA) *vs*. SV Y-composite grafts in OPCAB, the five-year occlusion rate and midterm clinical outcomes of the SV composite grafts were non-inferior to that of the RITA composite grafts. No significant differences between the two groups were found in overall survival and freedom from major adverse cardiac events at both five years and eight years. The use of an SV composite graft based on the in situ left ITA (LITA), as opposed to aortocoronary bypass graft, has numerous advantages. First, the SV composite graft is continuously exposed to protective effects of endothelium-derived mediators such as the nitric oxide released from the ITA. Furthermore, the length of the SV needed to reach the target vessel is shorter than that of an aortocoronary SV graft (SVG), and finally, the SV conduit anastomosed to the ITA is exposed to less circulatory stress than a conduit anastomosed to the ascending aorta^[33-36]^ (Figure 4).

The RA graft long-term patency was demonstrated to be higher in patients with progressively severe proximal stenoses, suggesting that RA grafting should not be considered in the setting of moderate (< 90% proximal obstruction) or questionably severe target vessel obstructions. Therefore, the benefit of RA graft patency over SVG patency is reliably seen when the degree of proximal coronary artery stenosis is severe (> 90%) and SVG should be preferred to revascularize the right coronary system when stenosis is < 90%^[37,38]^.

**Fig. 5:**
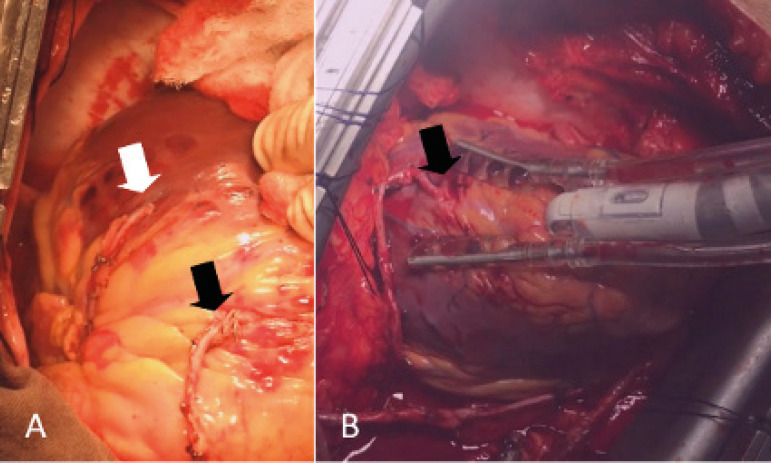
A. Left internal thoracic artery grafted to left anterior descending coronary artery (black arrow) and the in situ right internal thoracic artery (RITA) routed through the transverse sinus grafted to the obtuse marginal (OM) (white arrow). B. Free RITA grafted to OM (black arrow).

Long-term assessment of in situ and free RITA grafts patency revealed that the highest RITA failure rates were associated with grafting a native coronary artery with a stenosis of less than 60% compared with 80-100%. Grafts to non-left anterior descending coronary arteries had a greater risk of failure, the highest risk ratio being associated with grafting the right coronary artery (RCA). Preference should be given to grafting arteries with a high-grade stenosis or occlusion, to graft left rather than RCA, and in situ rather than free ITA grafts. Routing the RITA to the left side, either anterior to the aorta or through the transverse sinus, did not influence patency^[39]^ (Figure 5).

In patients where revascularization of the RCA was performed with SVG, with the right gastroepiploic artery, or RITA was used in a Y-composite fashion, the use of an SVG was associated with superior graft functionality compared with the other conduits and the graft function was negatively influenced by the minimum lumen diameter^[40]^.

Athanasiou et al., in a systematic review, aimed to identify if arterial grafts are superior to SVGs and whether graft failure rates vary between proximal and distal RCA anastomoses. Increased graft failure with the right gastroepiploic artery and RITA was found compared with SV. No significant difference was observed in late graft failure for RA compared with SV, although lower graft failure was observed with RA grafts to the proximal RCA when compared with SV^[41]^. A recent patient-level combined analysis of randomized, controlled trials performed by The Radial Artery Database International Alliance investigators failed to demonstrate a significant survival benefit despite superior patency of the RA graft compared with SVG^[36,42]^.

Performance of total arterial coronary revascularization has been promoted as offering the benefits of reduced morbidity and the need for reintervention with better long-term survival. However, the universal adoption rate remains extremely low, around 4-5% of total cases, because of relatively greater technical complexity, duration of the procedure, the perceived increased risk of sternal wound complications, biased patient selection, and lack of evidence from randomized controlled trials. Furthermore, an arterial conduit might not be the best one for grafting a less severely obstructed RCA^[18,36]^.

**Fig. 6:**
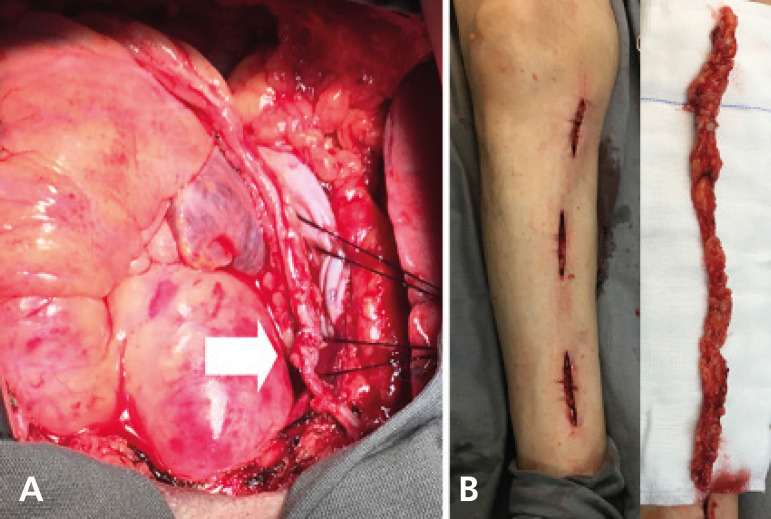
A. A no-touch saphenous vein grafted to the posterior descending coronary artery and proximally connected to the right internal thoracic artery stump (white arrow). B. The no-touch saphenous vein harvested through a minimally invasive technique.

Alternatively, a great improvement in outcomes has been achieved with SVG harvested with a no-touch (NT) technique. The NT technique of SV harvesting with an extensive pedicle of surrounding tissue, in which the manipulation and tension of the SV are minimized and manual intraluminal dilation is avoided during harvest, can potentially overcome the limitations of SVG when used as a composite graft based on the in situ LITA. Long-term patency of NT-SVGs seems less affected by coronary stenosis grade (a shortcoming for RA graft use), making this conduit preferable to RA grafts in CABG surgery, particularly for grafting the RCA^[43]^ (Figure 6).

## RITA STUMP AS GRAFT INFLOW SOURCE

The strategy of an SVG anastomosed to the ITA, rather than to the ascending aorta, can also be taken further and made more functional and physiological. Using the RITA proximal stump as a graft inflow source, as previously reported by Benussi et al.^[44]^, is a logical and straightforward surgical strategy, avoiding handling the aorta, theoretically granting further protection against neurological complications and stroke. Additionally, the caliber of the two vessels is similar and the wall thickness is comparable. No new skills are needed for applying the technique, at the reach of all competent surgeons routinely performing CABG. An additional Y-graft can be attached to the RITA stump itself or the connected vein, further expanding the scope of the anOPCAB (Table 1).

**Table 1:** Advantages of anastomosing the vein graft to the RITA proximal stump.

• No need for manipulation of the ascending aorta. • Higher production of nitric oxide through the RITA endothelium, with modulation of vascular tone and blood flow, and blunting platelet aggregation (thrombosis). • More physiological arterial pressure waveform, with less circulatory stress than a conduit anastomosed to the ascending aorta. • Wall thickness similarity between the grafts. • Better caliber match between the grafts.

RITA=right internal thoracic artery

## BILATERAL INTERNAL THORACIC ARTERY GRAFTING

Although the 10-year results of the Arterial Revascularization Trial (ART) showed no difference in survival between single ITA (SITA) or BITA grafts^[45]^, younger patients with fewer comorbidities stand to gain the most from BITA grafting and hence it should not be denied to this patient cohort. Noteworthy, based on observational evidence, the 2018 European Society of Cardiology/European Association for Cardio-Thoracic Surgery (ESC/EACTS) Guidelines on Myocardial Revascularization recommends the consideration of a second arterial graft (RITA or RA) as an adjunct to LITA in appropriate patients (class IIA)^[46]^.

In a recent multicenter cohort from the New Jersey Open Heart Surgery Registry with 42,714 patients undergoing CABG, with a median follow-up of 7.8 years, the long-term risks of MI and death associated with single-arterial CABG were higher than those associated with multiarterial CABG^[47]^. Benedetto et al. compared the outcomes of different conduit strategies in ONCAB and OPCAB surgeries in a single-center, observational study of 12,633 patients (1,818 with multiarterial OPCAB, 1,208 with multiarterial ONCAB, 4,412 with single-arterial OPCAB, and 5,195 with single-arterial ONCAB, considered the control group) undergoing primary isolated CABG surgery, with a mean follow-up of 8.2 years. The adjusted 30-day mortality and stroke were similar among the four groups, whereas the multiarterial OPCAB and ONCAB groups were associated with a significant fully adjusted 20% relative risk reduction in late mortality compared with standard single-arterial ONCAB. OPCAB was associated with a two-fold increased risk of incomplete revascularization irrespective of the use of multiarterial or single-arterial grafting. Multiarterial grafting is feasible in OPCAB and provides a long-term survival benefit compared with a conventional single-arterial strategy^[48,49]^.

## SKELETONIZED ITA

BITA harvesting has been associated with a higher incidence of deep sternal wound infections (DSWI), particularly in patients with diabetes or obesity^[50]^, and dissecting the ITA grafts with skeletonized technique results in a lower incidence of DSWI for BITA grafting^[51,52]^.

Using the Society of Thoracic Surgeons Adult Cardiac Surgery Database including 11,269 patients, the skeletonized BITA was associated with a decreased risk of DSWI, with no difference in operative mortality^[53]^.

Skeletonization increases the intraoperative flow and the length of the ITA by as much as 2.5 cm, allowing it to reach more distal targets and sequential grafting^[54,55]^.

## SURGEON AND TEAM EXPERIENCE AS A KEY FACTOR FOR OUTCOMES

Mounting evidence reveals that superior outcomes with OPCAB are associated with the surgeon and the operative team's experience and expertise. In an analysis of 2,094,094 patients undergoing ONCAB and OPCAB from the Nationwide Inpatient Sample database, OPCAB compared with ONCAB was associated with increased risk-adjusted mortality when performed in low-volume centers (< 29 cases/year) or by low-volume surgeons (< 19 cases/year). Conversely, in high-volume OPCAB centers (≥ 164 cases/year) and surgeons (≥ 48 cases/year), OPCAB reduced mortality compared with ONCAB in cases requiring a single graft or two or more grafts. Therefore, OPCAB outcome is dependent on volume at both the institution and individual surgeon levels and should not be performed at low-volume centers and by low-volume surgeons^[56]^.

A post hoc analysis of the ART demonstrate that surgeons experienced with both on-pump and off-pump techniques, whether using SITA or BITA grafts, yielded excellent results with no differences between the techniques, translated by low mortality, stroke, MI, and need for wound reconstruction and repeat revascularization^[57]^.

A recent large observational study revealed a reduction of mortality with off-pump compared with on-pump surgery in high-risk patients, regardless of the number of grafts, if performed by experienced surgeons^[58]^.

In a meta-analytic approach, Gaudino et al. demonstrated surgeons’ inexperience with the OPCAB procedure to be associated with increased mortality^[59]^. In surgical trials, a lack of experience and familiarity with the OPCAB will typically result in a high crossover rate to the control arm. The crossover rate from the OPCAB group was 7.9% and 9.7% in the CORONARY and the German Off-Pump Coronary Artery Bypass Grafting in Elderly Patients (GOPCABE) trials, respectively, *vs*. 12.4% in the ROOBY trial. In the largest randomized controlled trial meta-analysis of OPCAB *vs*. ONCAB to date (104 trials, 30,915 patients), a difference in survival in favor of ONCAB is found only in the studies with a crossover rate ≥ 10%, a rate that may be considered a surrogate marker for a lack of surgical proficiency with OPCAB techniques^[60]^

Saito et al., analyzing the Japan Cardiovascular Surgery Database, reported that isolated CABG was performed off-pump in 55.0% (n = 16,173) of all CABG cases (n = 29,392) in Japan. The operative mortality in elective cases was 1.1% for OPCAB compared with 2.5% for ONCAB, and all types of complications were lower for OPCAB than ONCAB, except for "readmission < 30 days"^[61]^.

Raja et al. compared the impact of OPCAB and ONCAB on short-term and long-term outcomes in a high-volume off-pump coronary surgery center in isolated first-time CABG procedures with at least two grafts; 5,995 OPCAB and 4,875 ONCAB were performed by surgeons with exclusive off-pump and on-pump practices. OPCAB performed by experienced surgeons, who perform only off-pump procedures in a high-volume off-pump coronary surgery center, was associated with a lower risk of operative deaths, fewer postoperative complications, and similar 20-year survival and freedom from reintervention rates compared with ONCAB^[62]^.

The 2018 ESC/EACTS Guidelines on Myocardial Revascularization state that OPCAB and preferably NT techniques on the ascending aorta by experienced operators is Class I recommendation in patients with significant atherosclerotic aortic disease. Also, Class IIa is granted to the technique for subgroups of high-risk patients. There is a special emphasis in patients with stable multivessel and/or left main coronary artery disease with porcelain aorta, where commonly the Heart Team recommendation is in favor of PCI unless expertise exists with an OPCAB. The guidelines recommend OPCAB in patients with renal impairment and suggest considering beating heart revascularization to reduce perioperative bleeding and the need for transfusions^[13,46]^.

## LONG-TERM OUTCOMES

The earlier concerns raised by the five-year follow-up of the ROOBY trial^[63]^, and also a 10-year analysis of a regional clinical registry in the United States of America^[64]^, suggesting an increased mortality and higher rate of graft failure in patients undergoing OPCAB were counterbalanced by a succession of well-conducted randomized controlled trials reporting long-term outcomes and demonstrating otherwise.

The MASS III trial was the first study to reach the longest ever follow-up at 10 years, with 308 patients randomized: 155 to OPCAB and 153 to ONCAB. The endpoints were freedom from death, MI, revascularization, and cerebrovascular events. No difference was found between the groups concerning primary composite endpoints at a 10-year follow-up. Although OPCAB surgery was associated with a lower number of grafts and a higher incidence of atrial fibrillation, it had no effects on long-term outcomes^[65]^.

The CORONARY trial randomized 4,752 patients to undergo OPCAB or ONCAB. The five-year outcome analyzed a composite outcome of death, stroke, MI, renal failure, or repeat coronary revascularization. No significant differences were seen between the off-pump group and the on-pump group in the rate of the composite outcome (23.1% and 23.6%, respectively)^[15]^.

The GOPCABE trial enrolled 2,539 patients aged ≥ 75 years who were randomly assigned to undergo OPCAB or ONCAB. The five-year follow-up data of this trial reported that 361 patients (31%) assigned to OPCAB and 352 patients (30%) assigned to ONCAB had died (hazard ratio OPCAB/ONCAB, 1.03; 95% confidence interval [CI], 0.89-1.19; *P*=0.71). The composite outcome of death, MI, and repeat revascularization occurred in 397 (34%) patients after OPCAB and in 389 (33%) patients after ONCAB (hazard ratio, 1.03; 95% CI, 0.89-1.18; *P*=0.704). Incomplete revascularization occurred in 403 (34%) patients in the OPCAB group and 354 (29%) in patients assigned to ONCAB (*P*<0.001). They concluded that in elderly patients ≥ 75 years of age, the five-year survival rates and the combined outcome of death, MI, and repeat revascularization were similar after ONCAB and OPCAB. Incomplete revascularization was associated with a lower five-year survival rate, irrespective of the type of surgery^[66]^.

These results reinforce the long-term follow-up of several other studies. No difference in mortality was seen in the Octopus trial after five years, BHACAS I and II trials after eight years, or in the SMART trial after eight years of follow-up^[67-69]^.

## LEFT THORACOTOMY AND MINIMALLY INVASIVE APPROACH

A left thoracotomy approach has been advocated as a safe alternative to a median sternotomy for CABG on the beating heart, allowing dissection and utilization of both ITA grafts for the left coronary system^[70]^.

Multivessel minimally invasive CABG, performed off-pump through a left anterolateral thoracotomy with BITAs has recently emerged as an alternative to conventional CABG, with low postoperative complications and encouraging outcomes^[71]^.

## EXCELLENCE CENTERS FOR CABG

The Excellence Centers for CABG have become an appealing concept, as specialization in surgical procedures has been shown to improve outcomes. Subspecialization in CABG and dedicated coronary surgery programs may lead to faster operations, increased use of BITA grafts, fewer complications, lower costs, and improved survival^[72]^.

These Excellence Centers would be able to implement OPCAB, minimally invasive, boost total arterial revascularization, adapt quickly to new techniques with proven advantages, and manage patients with CAD as part of a Coronary Revascularization Heart Team at high-quality, high-volume CABG hospitals^[73]^.

**Table:** 

Authors' roles & responsibilities	
WJG	Substantial contributions to the conception or design of the work; or the acquisition, analysis, or interpretation of data for the work; drafting the work or revising it critically for important intellectual content; final approval of the version to be published
ENG	Drafting the work or revising it critically for important intellectual content; final approval of the version to be published
ABJ	Drafting the work or revising it critically for important intellectual content; final approval of the version to be published
PHR	Drafting the work or revising it critically for important intellectual content; final approval of the version to be published
NAHJ	Drafting the work or revising it critically for important intellectual content; final approval of the version to be published
